# Ramipril attenuates lipid peroxidation and cardiac fibrosis in an experimental model of rheumatoid arthritis

**DOI:** 10.1186/ar4062

**Published:** 2012-10-18

**Authors:** Qin Shi, Jamilah Abusarah, Ghayath Baroudi, Julio C Fernandes, Hassan Fahmi, Mohamed Benderdour

**Affiliations:** 1 Orthopaedics Research Laboratory, Hôpital du Sacré-Cœur de Montréal and Department of Surgery, Université de Montréal, 5400 Gouin Blvd. West, Montreal, QC, H4J 1C5, Canada

## Abstract

**Introduction:**

Recent studies revealed that co-morbidity and mortality due to cardiovascular disease are increased in patients with rheumatoid arthritis (RA) but little is known about factors involved in these manifestations. This study aimed at characterizing the impact of arthritis on oxidative stress status and tissue fibrosis in the heart of rats with adjuvant-induced arthritis (AIA).

**Methods:**

AIA was induced with complete Freund's adjuvant in female Lewis rats. Animals were treated by oral administration of vehicle or angiotensin-converting enzyme inhibitor ramipril (10 mg/kg/day) for 28 days, beginning 1 day after arthritis induction. Isolated adult cardiomyocytes were exposed to 10 μM 4-hydroxynonenal (HNE) for 24 hours in the presence or absence of 10 μM ramipril.

**Results:**

Compared to controls, AIA rats showed significant 55 and 30% increase of 4-HNE/protein adducts in serum and left ventricular (LV) tissues, respectively. Cardiac mitochondrial NADP+-isocitrate dehydrogenase (mNADP-ICDH) activity decreased by 25% in AIA rats without any changes in its protein and mRNA expression. The loss of mNADP-ICDH activity was correlated with enhanced accumulation of HNE/mNADP-ICDH adducts as well as with decrease of glutathione and NADPH. Angiotensin II type 1 receptor (AT_1_R) expression and tissue fibrosis were induced in LV tissues from AIA rats. In isolated cardiomyocytes, HNE significantly decreased mNADP-ICDH activity and enhanced type I collagen and connective tissue growth factor expression. The oral administration of ramipril significantly reduced HNE and AT_1_R levels and restored mNADP-ICDH activity and redox status in LV tissues of AIA rats. The protective effects of this drug were also evident from the decrease in arthritis scoring and inflammatory markers.

**Conclusion:**

Collectively, our findings disclosed that AIA induced oxidative stress and fibrosis in the heart. The fact that ramipril attenuates inflammation, oxidative stress and tissue fibrosis may provide a novel strategy to prevent heart diseases in RA.

## Introduction

Rheumatoid arthritis (RA) is a common, systemic, autoimmune disease that leads to joint inflammation and progressive cartilage and bone erosion [1]. RA can also cause tissue inflammation around the joints as well as in other organs of the body [2]. Premature mortality among RA patients is frequently due to cardiovascular (CV) diseases and congestive heart failure (HF) [3,4]. In particular, given recent appreciation of the important role of inflammatory processes in the development and progression of atherosclerosis, interest has been focused on CV risk that might be associated with systemic inflammation in RA patients [5]. Abnormalities in the left ventricular (LV) structure and functions have also been reported in this population [6]. LV hypertrophy predicts CV events independently of traditional risk factors and therefore, if present, may also contribute to the early CV morbidity and mortality seen in RA patients [7]. This may provide a rationale for therapeutic interventions at an early stage of the disease process before overt CV disease has developed. In this regard, angiotensin-converting enzyme (ACE) inhibitors have been reported to improved endothelial function in patients with RA [8]. In addition to their effects on blood pressure, cardiac function, and antiproteinuric effect, ACE inhibitors have anti-inflammatory and immunomodulating properties [9]. However, much remains to be learned on the beneficial role of ACE inhibitors in preventing CV complications in RA patients.

Over the past 30 years, extensive experimental evidence has accumulated supporting the involvement of oxidative stress in the pathogenesis of RA and CV diseases [10-14]. Markers of oxidative stress contribute to and are correlated with disease activity in RA patients [15]. Oxidative stress contributes to chronic inflammation of tissues, plays a central role in dyslipidemia and atherosclerosis [16,17] and causes immunomodulation, which may lead to autoimmune diseases such as RA [18,19]. Furthermore, it contributes in T-cell activation that subsequently leads to endothelial dysfunction, decrease in endothelial progenitor cells and arterial stiffness, which are the congeners of accelerated atherosclerosis observed in RA patients.

One oxidative stress-related molecule that has generated considerable research interest over the past 10 years is 4-hydroxynonenal (HNE) [20]. HNE is an aldehyde end-product generated by peroxidation of the most abundant class of n-6 polyunsaturated fatty acids [21]. Similar to free radicals, aldehydes are electrophiles that react readily to nucleophilic residues of proteins, nucleic acids, and lipids, but their relatively longer half-life makes them candidates for the propagation of the damage to neighboring cells. The interest for HNE stems not only from its potential use as a biomarker of oxidative stress-induced lipid peroxidation (LPO), but also because of accumulating evidence indicating that HNE is able to modulate signaling pathways involved in cell proliferation, apoptosis, and inflammation, which are hallmarks of CV diseases [22,23]. However, much remains to be learned on the role of HNE as an active biomarker of oxidative stress-related events in RA patient that have an increased risk of developing CV diseases.

The principal aim of this study is to make a bridge between RA and cardiac complications by studying the involvement of HNE. Although accumulating evidence indicates that LPO products including HNE are increased and closely associated with RA, little data are available on the accumulation and the impact of this aldehyde in cardiac tissues in humans or animals with RA. Therefore, the present study aims to investigate the production of HNE in LV tissues from adjuvant-induced arthritis (AIA) rats and to identify a cellular target of HNE, namely the cardiac mitochondrial NADP+-dependent isocitrate dehydrogenase (mNADP-ICDH). We propose that accumulation of HNE in the heart may affect antioxidant status and cell metabolism and induce tissue fibrosis. In addition, we evaluated whether ramipril, an ACE inhibitor, reverses RA scores and prevents elevation of markers of oxidative stress, inflammation and tissue fibrosis.

## Materials and methods

### Adjuvant-induced arthritis (AIA) in rats

A total of 30 female Lewis rats (Charles River, Montreal, QC, Canada), weighing between 220 to 240 g, were studied. All animals were conditioned and manipulated according to Canadian Council on Animal Care guidelines. They were housed in pairs, in standard laboratory cages, and kept in an air-conditioned animal room at a temperature of 22°C ± 2°C and relative humidity of 57 ± 2% under a 12-h light/12-h dark cycle with *ad libitum *access to food and water. The experimental protocol was approved by the Research Ethics Board of Hôpital du Sacré-Cœur de Montréal. AIA was produced with complete Freund's adjuvant (CFA) containing heat-killed *Mycobacterium butyricum *(Difco, Detroit, MI, USA) in mineral oil at 10 mg/ml, as described previously [24]. The rats were injected intradermally with 100 μl of adjuvant at the base of the tail. Arthritis developed 10 days later. The rats were allocated to five groups, each consisting of six animals. Group 1, serving as control, received a 100 μl injection of mineral oil. Group 2 was given 10 mg/kg/day of ramipril alone by oral gavage. Group 3 was injected with 100 μl of CFA and represented the AIA model. Group 4 was given a 100 μl injection of CFA with 10 mg/kg/day of ramipril by oral gavage. Group 5 received a 100 μl injection of CFA with 10 mg/kg/day of 0.01% dimethyl sulfoxide (DMSO) as vehicle by oral gavage. Treatment was initiated on day 1 of the study and continued through day 28. All groups were sacrificed on day 28, when maximum inflammation occurred.

### Specimen selection

At the end of the study, the animals were anesthetized with xylazine/ketamine (10/50 mg/kg, intraperitoneally), and blood was collected in tubes by cardiac puncture before sacrifice. Blood samples were immediately centrifuged at 3,000 rpm for 10 min, and serum samples were stored at -80°C before they were used. LV tissues were isolated, freeze-clamped, and stored in liquid nitrogen until further analyses.

### Arthritis scoring

Arthritis severity was evaluated, as described previously [25], by monitoring the forepaws and hind paws twice a week for visual signs of inflammation, such as erythema and swelling, according to the following macroscopic scoring system. 0: no signs of arthritis, 1: swelling and/or redness in one paw, 2: two joints affected, 3: more than two joints affected, and 4: severe arthritis in all paws. Paw diameters were measured twice a week.

### Tissue levels of HNE-protein adducts

Total tissue levels of HNE-protein adducts were assessed in serum from animal groups by in-house enzyme-linked immunosorbent assay (ELISA), as described previously [26]. HNE-modified bovine serum albumin (BSA, Sigma-Aldrich, Oakville, ON, Canada) served as standard.

### Enzyme activity

Total mNADP-ICDH activity was quantified in 100 mg of powdered LV tissues that were homogenized on ice in 1 ml of lysis buffer composed of 25 mM Tris-HCl, pH 7.4, 150 mM NaCl, 1 mM EDTA, 1% NP-40 and 5% glycerol supplemented with protease inhibitor cocktail. The homogenate was then centrifuged for 10 min at 800 × *g *at 4°C. The supernatants were used for enzyme assay after 10-min centrifugation at 6,000 × *g *at 4°C. Protein levels were determined using bicinchoninic acid (BCA) protein assay reagent (Thermo Fisher Scientific, Rockford, IL, USA) with BSA (Sigma-Aldrich) as standard. The activity of mNADP-ICDH was assayed as the rate of NADP reduction (340 nm, ε = 6200 M ^-1^·cm^-1^) upon addition of 10 mM isocitrate, 1 mM NADP and 0.1 mM MgCl_2 _to 50 μg/ml of total proteins, as described previously [27]. Activities are expressed in units/mg of proteins, where 1 unit is defined as the amount of enzyme catalyzing the conversion of 1 μmol substrate/min at 37°C.

### Protein detection by Western blotting

Proteins from roughly 50 mg of powdered LV tissues were extracted, as described above. After protein determination, 20 μg of total proteins from heart tissues were subjected to discontinuous 4-12% sodium dodecyl sulfate-polyacrylamide gel electrophoresis (SDS-PAGE). Protein transfer and immunodetection as well as semi-quantitative measurements were taken, as outlined previously [27]. The primary antibodies were rabbit anti-mNADP-ICDH (Abcam, Cambridge, MA, USA), rabbit anti-angiotensin II receptor type 1 (AT_1_R) (Pierce Biotechnology, Inc., Rockford, IL, USA), anti-β-actin (Santa Cruz Biotechnology, Santa Cruz, CA, USA), anti-HNE (Cayman Chemical Company, Ann Arbor, MI, USA), and anti-type I collagen (Col I) and anti-connective tissue growth factor (CTGF) (Millipore, Etobicoke, ON, Canada). After serial washes with Tris-buffered saline + Tween, primary antibodies were detected by goat anti-rabbit IgG conjugated with horseradish peroxidase (Cell Signaling Technology, Danvers, MA, USA). Immunoreactive proteins were quantified with SuperSignal blotting substrate (Thermo Fisher Scientific) and exposed to clear-blue X-ray film (Thermo Fisher Scientific).

### Immunoprecipitation

For the *in vivo *detection of HNE/mNADP-ICDH adducts, 100 μg of total protein, prepared as indicated above, were subjected to overnight immunoprecipitation with 1 μg of rabbit anti-mNADP-ICDH (Abcam) in lysis buffer at 4°C with constant, gentle shaking and then for 2 more hours with protein A (Santa Cruz Biotechnology), as described previously [27]. The resin was washed with lysis buffer, and proteins were removed from the resin by the addition of 100 μl undiluted SDS-loading buffer. A 20-μl aliquot of immunoprecipitated proteins was heated at 95°C for 3 min prior to Western blot analysis, with rabbit anti-HNE as primary antibody (1:1,000 dilution; Cayman Chemical Company).

### RNA extraction and reverse transcription-polymerase chain reaction (RT-PCR)

Total RNA was isolated with TRIzol reagent according to the manufacturer's instructions (Invitrogen, Burlington, ON, Canada). RNA was measured with RiboGreen RNA quantitation kits (Molecular Probes, Eugene, OR, USA), dissolved in diethylpyrocarbonate-treated H_2_O, and stored at -80°C until used. One μg of total RNA was reverse-transcribed with Moloney murine leukemia virus reverse transcriptase (Fermentas, Burlington, ON, Canada), as detailed in the manufacturer's guidelines. One-fiftieth of the reverse transcriptase reaction product was analyzed by traditional PCR or real-time quantitative (q) PCR [28]. The following sense and anti-sense specific primers (Bio-Corp, Inc., Montreal, QC, Canada), were tested: rat mNADP-ICDH (forward) 5'-ATG TGG AAG AGC CCT AAC GGA ACT-3', (reverse) 5'-ACA TGC CAG CTC GAT CTA CCA CAA-3'; rat AT_1_R (forward) 5'-CGG CCT TCG GAT AAC ATG AG-3', (reverse) 5'-CCT GTC ACT CCA CCT CAA AAC A-3'; rat GAPDH (forward) 5'-GCA TTG ATG GTG AGG TGA GCA AA-3', (reverse) 5'-TCG CTC CTG GAA GAT GGT GA-3'. qPCR analysis was performed in a total volume of 50 μl containing template DNA, 200 nM sense and anti-sense primers, 25 μl of SYBR Green Master Mix (Qiagen, Mississauga, ON, Canada), and 0.5 units of uracil-*N*-glycosylase (UNG; Epicentre Technologies, Madison, WI, USA). After incubation at 50°C for 2 min (UNG reaction) and at 95°C for 10 min (UNG inactivation and activation of AmpliTaq Gold enzyme), the mixtures were subjected to 40 amplification cycles (15 s at 95°C for denaturation and 1 min for annealing and extension at 60°C). SYBR Green dye incorporation into the PCR products was monitored in real time with a Mx3000 real-time PCR system (Stratagene, La Jolla, CA, USA), to determine the threshold cycle (C_t_) at which exponential amplification of PCR products begins. After PCR, dissociation curves were generated with 1 peak indicating amplification specificity. A C_t _value was obtained from each amplification curve with software provided by the manufacturer (Stratagene).

Relative mRNA expression in chondrocytes was quantified according to the ΔΔC_t _method, as detailed in the manufacturer's guidelines (Stratagene). A ΔC_t _value was first calculated by subtracting the C_t _value for the housekeeping gene GAPDH from the C_t _value for each sample. A ΔΔC_t _value was then calculated by subtracting the ΔC_t _value for the controls (unstimulated cells) from the ΔC_t _value for each treatment. Fold changes compared to the controls were then quantified by 2^-ΔΔC^_t_. Each PCR generated only the expected specific amplicon, as shown by melting temperature profiles of the final product and gel electrophoresis of the test PCRs. Each PCR was performed in triplicate on two separate occasions for each independent experiment.

### Prostaglandin E2 (PGE_2_) and tumour necrosis factor-alpha (TNFα immunoassay

PGE_2 _and TNFα levels were measured in serum by enzyme immunoassay or ELISA, with kits from the Cayman Chemical Company and R&D Systems (Minneapolis, MN, USA), respectively. Detection sensitivity was 9 pg/ml for PGE_2 _and 4.4 pg/ml for TNFα. Each assay was run according to the manufacturer's specifications.

### Evaluation of tissue fibrosis by picrosirius red staining

Tissue samples embedded in Tissue Fix were transferred after 24 h in a formalin-ethanol solution (50/50%) until they were sliced longitudinally. They were then stained with picrosirius red (Sigma-Aldrich) and examined under circularly polarized light to visualize interstitial collagen from its red coloration [29]. Photographs were taken, and the surface staining for collagen was calculated as a percentage of the total surface with the use of the National Institutes of Health program ImageJ.

### Cardiomyocytes isolation

Adult cardiomyocytes were isolated from female Lewis rats, as we described previously [30]. The resulting hydrolysate was filtered to remove cell clumps and depleted of fibroblasts by two 35-min preplatings at 37°C, in Dulbecco's modified Eagle's minimal essential medium (DMEM, Invitrogen) containing 10% fetal bovine serum (FBS, Invitrogen). The non-adhered cardiomyocyte suspension was centrifuged, resuspended in DMEM + 10% FBS, seeded in 12-well plates at an approximate density of 2 × 10^5 ^cells/cm^2^, and incubated for 24 h at 37°C in an atmosphere of 5% CO_2_. The spent medium was replaced by serum-free DMEM medium supplemented with the following products supplied by Sigma-Aldrich: 1% BSA, 1× vitamins, 1× nonessential amino acids, 0.5× essential amino acids, 6.25 μg/ml insulin, 6.25 μg/ml transferrin, 6.25 ng/ml sodium selenite, 80 μg/ml CaCl_2_, 50 U/ml penicillin, 50 μg/ml streptomycin, and 20 μM cytosine-β-D-arabinofuranoside. The culture was maintained at 37°C for 48 h in a humidified atmosphere containing 5% CO_2_/95% air in the presence or absence of 10 μM HNE and/or 10 μM ramipril.

### Quantification of reduced glutathione (GSH), oxidized glutathione (GSSG) and NADPH levels

Powdered LV tissues (50 mg) were homogenized on ice in 1 ml of lysis buffer containing 0.2 M 2-(N-morpholino)ethanesulfonic acid, 0.05 M potassium phosphate, and 1 mM EDTA, pH 6.0. The homogenates were then centrifuged for 10 min at 800 × *g *at 4°C followed by another centrifugation for 10 min at 10,000 × *g *at 4°C. The protein content of tissue extracts was determined using BCA protein assay reagent (Pierce), with BSA (Sigma-Aldrich) as standard. GSH and NADPH levels were quantified with a GSH Assay Kit (Cayman Chemical Company) and NADP/NADPH Assay Kit (Abcam) according to the manufacturers' directions. The quantification of GSSG was accomplished by derivatizing GSH by adding vinylpyridine to total proteins. Values were expressed as GSSG/(GSSG+GSH) and NADP/(NADP+NADPH) ratios.

### Statistical analysis

The data are expressed as means ± SEM of six rats. All statistics were generated by Prism software (GraphPad Software, San Diego, CA, USA). Statistical significance was assessed by one-way ANOVA, followed by the Bonferroni multiple-comparison post test, and *P *< 0.05 was considered significant.

## Results

### Serum TNFα and PGE_2 _levels and clinical evaluation

**Figure 1:**
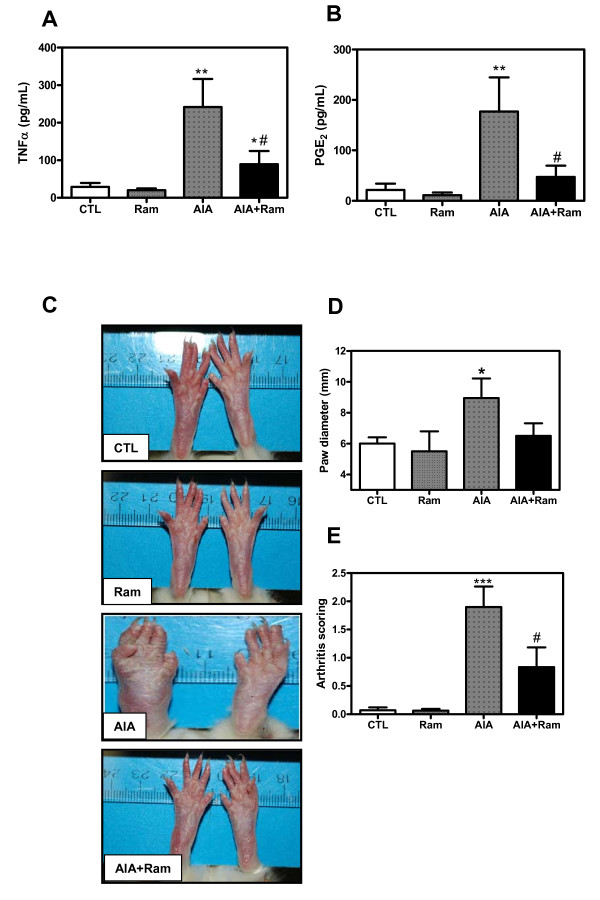
**Pro-inflammatory cytokines determination and clinical evaluation of adjuvant-induced arthritis (AIA)**. **(A) **Tumor necrosis factor-alpha (TNFα) and **(B) **prostaglandin E2 (PGE_2_) were assessed in serum from control and AIA rats with ELISA. **(C-E) **Clinical evaluation was performed by measuring paw swelling (C, D) and arthritis score (C, E). Data are mean ± SEM of three experiments. Statistics: one-way ANOVA, *P < 0.05, **P < 0.01, ***P < 0.001 (CTL versus AIA), ^#^*P *< 0.05 (AIA versus AIA+Ram). CTL, control; Ram, ramipril.

First, we tested ramipril ability to reduce inflammation and arthritis markers in our experimental RA model. Serum TNFα and PGE_2 _levels were measured to evaluate the extent of inflammation process. Hind paw swelling and arthritis scores were recorded. These markers reflect both inflammatory and arthritic changes occurring in rats with AIA. As shown in Figure 1, TNFα (Figure 1A) and PGE_2 _(Figure 1B) levels were significantly higher in rats with AIA (approximately 8-fold increase, *P *< 0.01) than in the controls. In comparison to arthritic animals, however, ramipril-treated rats displayed a significant decrease in serum TNFα and PGE_2 _levels (approximately 2.6-fold diminution, *P *< 0.05). Furthermore, our data revealed that oral ramipril administration to adjuvant-immunized rats reduced the progression of arthritis by inhibiting the increase in paw swelling (Figures 1C and 1D) and arthritis score (Figures 1C and 1E) compared to arthritic rats. None of the indicated parameters including TNFα, PGE_2 _and arthritis scoring was changed in control AIA rats receiving DMSO (data not shown) or control animals receiving ramipril alone.

### The LPO end-product HNE is increased in serum and LV tissues of AIA rats

**Figure 2:**
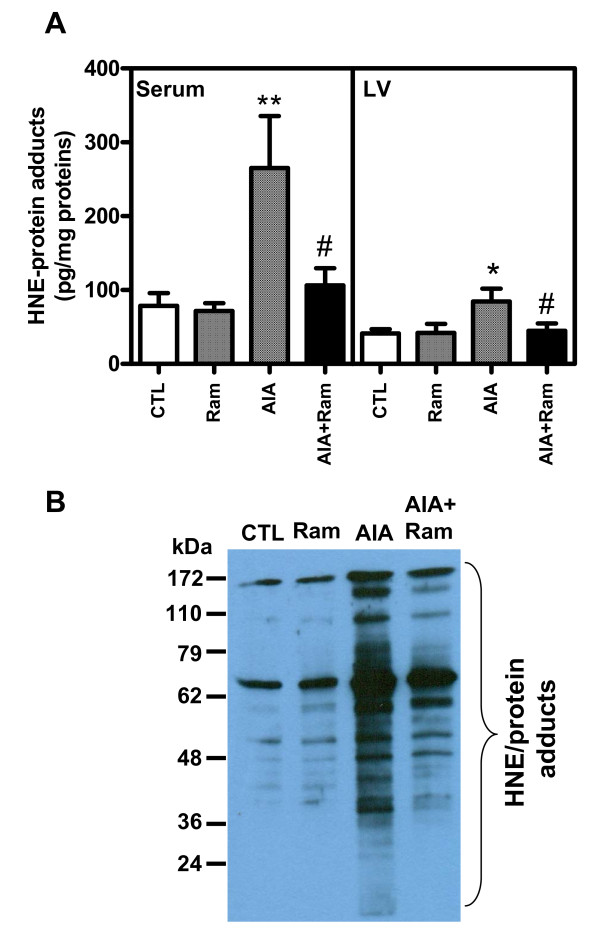
**Quantification and characterization of 4-hydroxynonenal (HNE)/protein adducts by enzyme-linked immunosorbent assay (ELISA) and Western blotting**. **(A) **HNE/protein adducts were measured by ELISA in serum and left ventricular (LV) tissues from control and adjuvant-induced arthritis (AIA) rats (*n *= 6). **(B) **HNE/protein adducts were characterized by Western blotting in total proteins of LV tissues from control and AIA rats. Values are the mean ± SEM of three experiments. *P < 0.05; **P < 0.01 (CTL versus AIA), ^#^*P *< 0.05 (AIA versus AIA+Ram) by one-way ANOVA. CTL, control; Ram, ramipril.

Here, we looked for evidence of increased oxidative stress in LV tissues from AIA rats, by quantification of HNE, a very reactive product of LPO, in both serum and LV tissues. As illustrated in Figure 2A, HNE levels rose in the serum and LV tissues of AIA rats by 3.3- (*P *< 0.01) and 2-fold (*P *< 0.05) respectively compared to control rats. The corresponding profile of HNE/protein adducts generated in LV tissues is charted in Figure 2B. As can be seen, the intensity of immunoreactive bands increased in LV tissues from AIA rats in comparison to controls, and most had a molecular weight ≥40 kDa. Interestingly, the accumulation of cardiac HNE/protein adducts was significantly abolished by ramipril administration in both serum and LV tissues (*P *< 0.05). No significant changes in HNE production was noted in control AIA rats receiving DMSO (data not shown) or control animals receiving ramipril alone (Figure 2).

### Cardiac mNADP-ICDH is impaired in AIA rats

**Figure 3:**
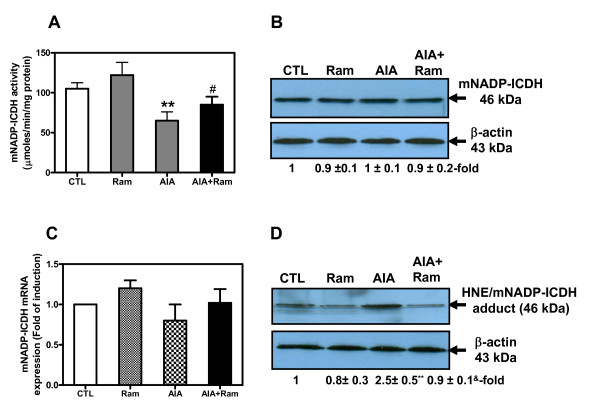
**Cardiac mitochondrial NADP+-dependent isocitrate dehydrogenase (mNADP-ICDH) activity (A), protein (B), and mRNA (C)**. Total proteins and RNA were extracted from left ventricular (LV) tissues of control and adjuvant-induced arthritis (AIA) rats and processed to enzymatic assay **(A)**, Western blotting **(B) **or real-time PCR **(C)**, as described under Materials and Methods. **(D) **Cardiac 4-hydroxynonenal (HNE)/NADP-ICDH adducts were identified in total proteins by immunoprecipitation as described in Material and Methods. Values are the mean ± SEM of three experiments. **P < 0.01 (CTL versus AIA), ^#^*P *< 0.05, ^&^*P *< 0.01 (AIA versus AIA+Ram) by one-way ANOVA. CTL, control; Ram, ramipril.

To establish whether mitochondrial bioenergetics/redox status were impaired in the heart of AIA rats, we assessed the activity and the expression of mNADP-ICDH, an enzyme whose activity is a determinant of mitochondrial energy and oxidative stress status. We observed that mNADP-ICDH activity decreased by 25% (*P *< 0.01, Figure 3A) in LV tissues. In contrast, its protein (Figure 3B) and mRNA (Figure 3C) expression levels remained unchanged.

We then tested the hypothesis of potential mNADP-ICDH modification by HNE. Our choice of HNE is based on previous reports indicating that mNADP-ICDH is targeted for HNE binding in heart hypertrophy in spontaneously hypertensive rats (SHR) [27]. Data derived by the immunoprecipitation approach, using rabbit anti-mNADP^+^-ICDH antibody followed by Western blotting with rabbit anti-HNE antibody, showed a 2.5-fold (*P *< 0.01) increase in relative HNE/mNADP-ICDH adduct levels in heart tissues from AIA rats compared to controls (Figure 3D). Interestingly, all changes in this enzyme were prevented by oral treatment with ramipril. However, the administration of DMSO (data not shown) or ramipril had no effect on mNADP-ICDH activity/expression in AIA and control rats, respectively. Taken together, our results indicate the existence of a post-translational mechanism decreasing cardiac mNADP-ICDH activity during the RA process.

### Alteration of cardiac redox status in AIA rats

**Figure 4:**
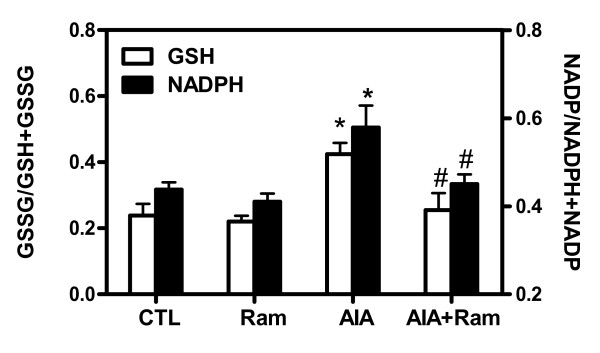
**Determination of cardiac redox status**. Glutathione (GSH) and nicotinamide adenine dinucleotide phosphate (reduced) (NADPH) were determined in total proteins extracted in left ventricular (LV) tissues from control and adjuvant-induced arthritis (AIA) rats. Data are mean ± SEM of three experiments and expressed as GSSG/(GSSG+GSH) and NADP/(NADP+NADPH) ratio. Statistics: one-way ANOVA; *P < 0.05 (CTL versus AIA), ^#^*P *< 0.05 (AIA versus AIA+Ram). CTL, control; Ram, ramipril.

Because impairments of mNADP-ICDH activity/expression were demonstrated to be associated with alterations in oxidative stress status [31], we evaluated whether changes in this enzyme were paralleled by similar modifications of NADPH, a co-factor involved in GSH generation. Figure 4 depicts a significant 30% decrease (*P *< 0.05) of NADPH and GSH pools in LV tissues of AIA rats compared to controls. The impairment of cardiac redox status was restored by ramipril. No significant changes in redox status were apparent in AIA rats receiving DMSO (data not shown) or control animals receiving ramipril alone. Collectively, these findings suggest that alteration of myocardial redox status could be due to mNADP-ICDH inactivation.

### Cardiac fibrosis was increased in AIA rats

**Figure 5:**
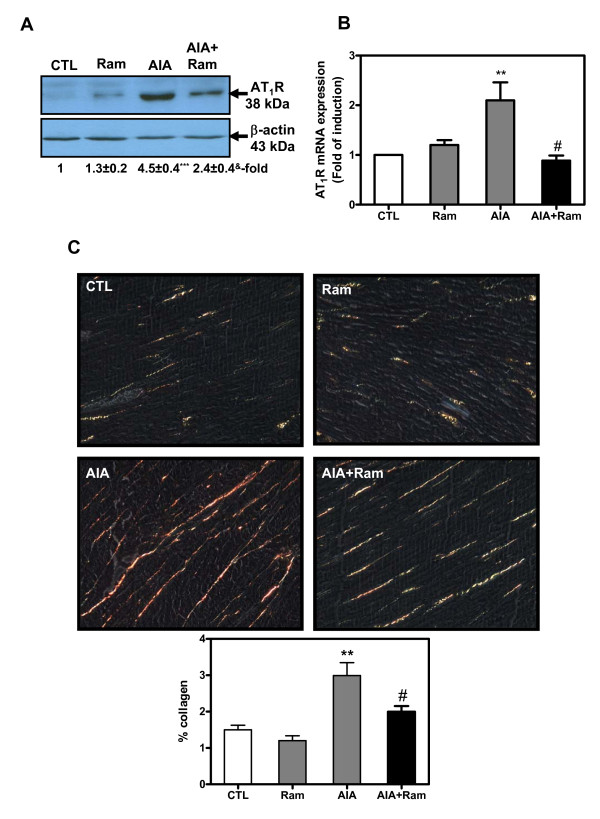
**Measurement of cardiac remodeling in left ventricular (LV) tissues from control and adjuvant-induced arthritis (AIA) rats**. **(A, B) **Expression of angiotensin II type I receptor (AT_1_R) LV from AIA rats. Total proteins and RNA were extracted in LV tissues of control and AIA rats and processed to AT_1_R protein (A) and mRNA (B) determination by Western blotting and real-time PCR, respectively. **(C) **Cardiac collagen content in LV tissues was detected by the picosirius red polarization method. Histological sections obtained from control and AIA rats were stained with picosirius red solution and examined under circularly polarized light to visualize interstitial collagen by its red coloration. Surface staining for collagen was calculated as a percentage of the total surface. Values are the mean ± SEM of three experiments. **P < 0.01, ***P < 0.001 (CTL versus AIA), ^#^*P *< 0.05, ^&^*P *< 0.01 (AIA versus AIA+Ram) by one-way ANOVA. CTL, control; Ram, ramipril.

First, a pair of experiments was designed to verify the expression of heart fibrosis markers in AIA rats. Our findings illustrated in Figure 5 showed that AT_1_R protein (Figure 5A) and mRNA (Figure 5B) levels were respectively 4.5- (*P *< 0.001) and 2.1-fold (*P *< 0.01) higher in LV tissues from AIA rats as compared to controls. Ramipril attenuated the elevation of AT_1_R protein and mRNA expression in agreement with the drug's capacity to prevent cardiac hypertrophy [32]. Altogether, our data suggested that increased tissue AT_1_R expression could potentiate heart hypertrophy and hyperplasia in AIA rats.

Second, another set of experiments were performed to document cardiac fibrosis in each animal group. As illustrated in Figure 5C, when microscopic images prepared from each groups were compared, a significant increase of collagen content (calculated as a percent of total surface) was observed in LV tissues from AIA rats as compared to controls (CTL: 1.5 ± 0.12, RA: 2.9 ± 0.35, **P < 0.01). However, the oral administration of ramipril significantly reduced the collagen deposition in the tissue (1.9 ± 0.14, ^#^*P *< 0.05). No significant changes in cardiac fibrosis were noted in AIA rats receiving DMSO (data not shown) or control animals receiving ramipril alone.

### HNE induced mNADP-ICDH inactivation as well as Col I and CTGF expression *in vitro*

**Figure 6:**
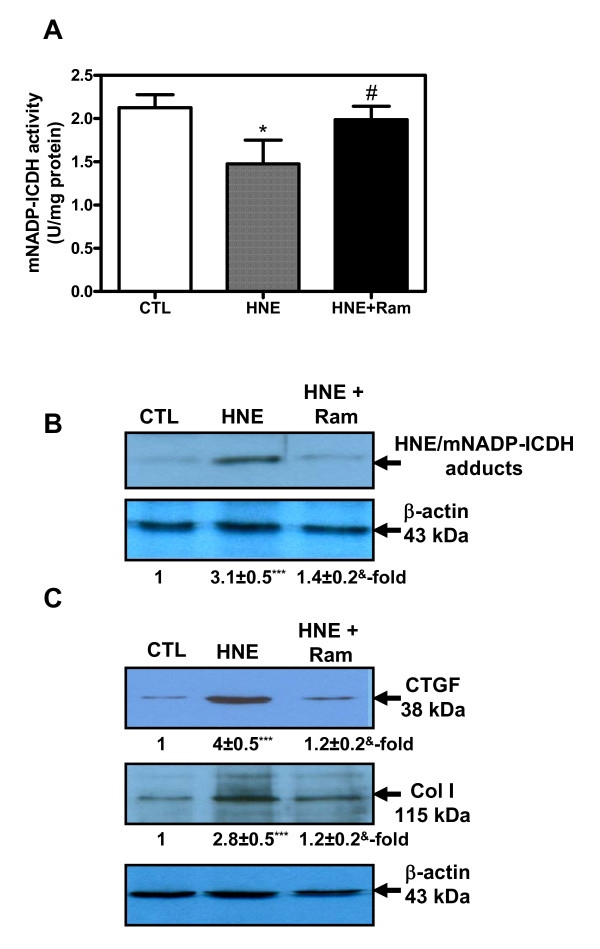
**Modulation of mitochondrial NADP+-dependent isocitrate dehydrogenase (mNADP-ICDH), type I collagen (Col I) and connective tissue growth factor (CTGF) in isolated adult rat cardiomyocytes**. Cells were treated with 10 μM 4-hydroxynonenal (HNE) for 48 hours in the presence or absence of 10 μM ramipril and then total proteins were preceded to the determination of **(A) **NADP-ICDH activity, **(B) **HNE/NADP-ICDH adducts, and **(C) **Col I and CTGF protein as described in Materials and Methods. Values are the mean ± SEM of three experiments. *P < 0.05, ***P < 0.001 (CTL versus HNE), ^#^*P *< 0.05, ^&^*P *< 0.01 (HNE versus HNE+Ram) by one-way ANOVA. CTL, control; Ram, ramipril.

To confirm the *in vivo *observation, we conducted a series of *in vitro *experiments that mimicked the *in vivo *situation during RA development. First, we investigated the effect of HNE on mNADP-ICDH in isolated rat cardiomyocytes. As illustrated in Figure 6, treatment of cells with 10 μM HNE for 48 h reduced mNADP-ICDH activity by approximately 30% (*P *< 0.05, Figure 6A) and increased HNE/mNADP-ICDH adducts by 3.1-fold (*P *< 0.001, Figure 6B). To evaluate the ability of HNE to induce markers of fibrosis, cells were treated in the indicated conditions. Figure 6C showed that HNE induced Col I and CTGF by 4- (*P *< 0.001) and 2.8-fold (*P *< 0.001), respectively. Nevertheless, all HNE effects were abolished by 10 μM ramipril. Collectively, these findings are in agreement with the *in vivo *results and confirm the potential role of HNE in mNADP-ICDH inactivation and fibrosis induction in the heart.

## Discussion

The present study provides clear evidence of increased cardiac fibrosis and oxidative stress status in an experimental rat model of RA. This model is robust, the incidence rate of the disease is 100%, and AIA in rats shares many features with RA in humans, such as inflammation, marked bone resorption and periosteal bone proliferation [33]. AIA in rats has been widely adopted as an experimental paradigm for the pre-clinical screening of RA treatments. In this model, we measured markers of RA. Our data showed an increase of paw diameter, arthritis scores and inflammatory mediator levels in serum known to be involved in the RA process, such as TNFα and PGE_2_, in AIA rats. These findings are in agreement with our previous study indicating an elevation of these pro-inflammatory mediators in serum from AIA rats [24]. In RA, fibroblast-like synoviocytes and inflammatory cells produce a panoply of pro-inflammatory mediators, such as IL-1β, TNFα, and PGE_2_, which play key roles in the pathogenesis of RA. They promote inflammation, hyperplasia, and bone and cartilage destruction [34].

In view of its potential pathophysiological significance, we first investigated oxidative stress status in heart tissues. Reactive oxygen species (ROS), which likely contribute to the pathophysiology of endothelial dysfunction, were shown to be overproduced in the aorta of rats with AIA [35]. ROS can damage cellular components by direct oxidation; however, given the susceptibility of polyunsaturated fatty acids to oxidative attacks, there is a strong rationale for invoking the involvement of secondary products of LPO, such as HNE and acrolein, in ROS-related injury. Our data disclosed an increase of HNE levels in both serum and heart tissues from AIA rats. These observations are in agreement with previous data reported by Choi [2], showing an increase of malondialdehyde, protein carbonyl and advanced glycation end-products in the heart of AIA rats. The fact that activation of HNE-detoxifying enzymes protects the heart from ischemic injury suggests that HNE is a significant contributor to myocardial damage [36]. Many studies have shown that HNE damages electron transport chain complexes [37-39], affecting both cell respiration and critical events, such as calcium-induced permeability transition [40]. Considering these results, it is likely that extensive formation of HNE/protein adducts causes damage to mitochondrial energy metabolism that culminates in altered heart function. Increased oxidative stress and/or changes of fuel metabolism are among factors that contribute to the development of cardiac hypertrophy and its progression to HF, regardless of disease etiology [41].

Second, our findings revealed that mNADP-ICDH activity was decreased in heart tissues from AIA rats without changes in its expression at protein and mRNA levels. Inactivation of this enzyme was associated with the formation of HNE/mNADP-ICDH adducts. In our previous study, we reported elevated HNE/protein adducts levels in SHR compared to Wistar-Kyoto rat hearts [27]. Specifically, the post-translational modification of mNADP-ICDH by HNE precedes cardiac hypertrophy development. In addition to its critical role in the regulation of mitochondrial energy, mNADP-ICDH has received considerable attention as a principal source of NADPH, a co-factor involved in GSH regeneration [31]. The generation of GSH by GSH reductase increases the mitochondrial capacity for HNE detoxification through GSH conjugation and export [42]. In the present study, we found a decrease in NADPH and GSH pools, probably attributed to mNADP-ICDH inactivation. We speculate that in the heart, where mNADP-ICDH favors the generation of α-ketoglutarate and NADPH, mNADP-ICDH inactivation by HNE could compromise mitochondrial energy metabolism and redox status.

To confirm the *in vivo *data, we conducted additional experiments on isolated adult rat cardiomyocytes treated with HNE, an *in vitro *approach designed to mimic the *in vivo *situation during RA process. Interestingly, results revealed that HNE inhibits mNADP-ICDH activity and augments HNE/mNADP-ICDH adduct levels, indicating an impairment of mitochondrial function. The mechanism causing this inactivation could reside in HNE ability to bind to a cysteine residue near the substrate's binding site [27]. In another study, it has been reported that nitric oxide (NO) inhibits the activity of mNADP-ICDH [43], through S-nitrosylation of cysteine residues on this enzyme. The NO-mediated damage to mNADP-ICDH results in the perturbation of the cellular antioxidant defense mechanisms and subsequently leads to a pro-oxidant condition. Taken together, our data provide direct *in vivo *and *in vitro *evidence that oxidative stress contributes to mNADP-ICDH inactivation in conjunction with the formation of HNE/mNADP-ICDH adducts. Additional work is needed, however, to evaluate the specific significance of mNADP-ICDH inactivation (via HNE binding) in cardiac dysfunction in RA.

Third, our data showed that cardiac fibrosis is higher in AIA rats. On one hand, we demonstrated an increase of tissue fibrosis in LV tissues of AIA rats. Fibrosis is a hallmark of CV diseases and is often associated with inflammation. It has been proposed that mediators of inflammation, including cytokines such as TNFα, IL1β, and IL-6, leads to microvascular dysfunction and ultimately to myocardial remodeling and fibrosis [44,45].

Augmented fibrosis in the heart is linked with ventricular remodeling and subsequent cardiac dysfunction and is therefore a common pathological feature of many types of HF [46]. Traditionally, cardiac fibroblasts were thought to be activated by pro-inflammatory processes to proliferate and synthesize collagen that is secreted and deposited in the interstitial space [47]. Transgenic mice with targeted overexpression of TNFα develop progressive myocardial fibrosis, diastolic dysfunction, and adverse cardiac remodeling [48]. Furthermore, it has been reported that cardiac remodeling was markedly exacerbated in mice that express high level of human C-reactive protein (CRP), resulting in a significant reduction in the LV ejection fraction and fractional shortening and an increase in cardiac fibrosis (Col I and III) and inflammation (IL-1β and TNFα). The enhancement in cardiac remodeling in mice that express human CRP was associated with further upregulation of the AT_1_R [49].

Furthermore, the greater increase of HNE levels in the heart of AIA rats would play a key role in the pathogenesis of fibrosis. In isolated cardiomyocytes, we revealed the capacity of HNE to induce Col I and CTGF. These *in vitro *data suggest that HNE may act as a potent pro-fibrogenic stimulus in the heart. In fact, many studies have shown that LPO products play a key role in the initiation and progression of fibrosis in different organs [50]. During liver injury, Zamara *et al. *[51] have indicated that HNE favor extracellular matrix deposition by its ability to upregulate Col I and tissue inhibitors of metalloproteinase-1 (TIMP-1) expression. On the other hand, we noted a significant increase of AT_1_R in heart tissues from AIA rats. These findings are consistent with those reported by Sakuta *et al. *[52], who demonstrated that AT_1_R and ACE expression was heightened in the aortas of rats with AIA, suggesting the involvement of renin-angiotensin system (RAS) in vascular damage in an animal model of autoimmune/inflammatory disease. The mechanism underlying the upregulation of AT_1_R in RA remains unclear. It has been shown that circulating pro-inflammatory cytokines influence the vascular expression of AT_1_R. IL-6 is of particular interest, since it has been demonstrated to induce the upregulation of AT_1_R gene and protein expression in rat cultured vascular smooth muscle cells and in mouse vascular tissue [53].

Finally, our findings of enhanced markers of cardiac oxidative stress and fibrosis as well as arthritis scoring were prevented by ramipril administration in AIA rats. It is well established that RAS activation by an agonist of AT_1_R results in cardiac fibrosis, inflammation, oxidative stress and congestive heart failure via AT_1 _receptor activation [54]. The latter mediates effects such as vasoconstriction, cellular proliferation, and matrix deposition [55,56], most likely via a mechanism that involves enhanced free radical production due to stimulated NADPH oxidase activity [57,58]. Angiotensin II (Ang II) stimulates the expression of NF-κB, a transcription factor that regulates gene expression of inflammatory cytokines and surface adhesion molecules, leading to inflammation [59]. NF-κB activation may result from increased oxidative stress, which may be induced by Ang II [60]. Interestingly, it has been reported that Ang II-induced myocardial molecular/cellular events and their relation to oxidative stress, inflammation and fibrosis are attributed, in part, to the production of mineralocorticoids such as aldosterone [61]. It also produces a variety of other actions that lead to progressive damage in the heart, vasculature, and kidneys [62,63]. Although Ang II is considered to be the major mediator of cardiovascular damage, it was suggested that aldosterone may mediate and exacerbate the effects of Ang II [58,61]. Blocking of the mineralocorticoid receptor and inhibition of ACE and/or the AT_1_R reduced aldosterone release and markers of fibrosis and oxidative stress.

The use of ACE inhibitors in patients with RA has attracted much interest in recent years. Flammer *et al. *[8] reported that ramipril markedly improved endothelial function in RA patients, by reducing the levels of pro-inflammatory cytokines. However, arthritis activity score remained unaltered after treatment with ramipril. These data contrast with those reported by Tikiz *et al. *[64] which showed no significant changes in vasodilatation, CRP and TNFα in RA patients treated with quinapril. The authors attributed the lack of quinapril efficacy to improper dosage or length of treatment. In an experimental model of RA, Dalbeth and colleagues [65] observed that the ACE inhibitor quinapril and the AT_1_R inhibitor candesartan suppressed the severity of collagen-induced arthritis. Interestingly, they noted that paw TNFα concentration was reduced in mice receiving quinapril compared to those administered water and that decreased TNFα levels were not a consequence of suppressed disease activity. More recently, Sakuta *et al. *[52] noted an increase in AT_1_R and ACE expression in the aortas of rats with AIA. In addition to ACE inhibitors, the administration of AT_1_R blockers decreased the oxidative stress as measured by superoxide generation and NADPH oxidase expression in the aortas of rats with AIA [52].

## Conclusions

Our findings showed for the first time that myocardial abnormalities are observed in AIA rats. These include increase in oxidative stress and tissue fibrosis and alteration in cell metabolism. The findings also inform new hypothesis for the role of HNE in myocardial abnormalities through its ability to inhibit mNADP-ICDH activity and to induce CTGF and Col I expression in isolated cardiomyocytes. Potentially, it could contribute to the etiology of cardiomyopathy. Furthermore, our data suggest that ACE inhibition may provide a novel strategy to prevent CV events in RA.

## Abbreviations

ACE: angiotensin-converting enzyme; AIA: adjuvant-induced arthritis; Ang II: angiotensin II; AT_1_R: angiotensin II receptor type 1; BSA: bovine serum albumin; Col I: type I collagen; CFA: complete Freund's adjuvant; CRP: C-reactive protein; CTGF: connective tissue growth factor; CV: cardiovascular; DMEM: Dulbecco's modified Eagle's medium; ELISA: enzyme-linked immunosorbent assay; FBS: fetal bovine serum; GSH,: glutathione; HF: heart failure; HNE: 4-hydroxynonenal; IL-1β: interleukin-1beta; kDa: kiloDalton; LPO: lipid peroxidation; LV: left ventricular; mNADP-ICDH: mitochondrial NADP+-isocitrate dehydrogenase; PGE_2: _prostaglandin E2; qPCR: quantitative polymerase chain reaction; RA: rheumatoid arthritis; RAS: rennin-angiotensin system; ROS: reactive oxygen species; SHR: spontaneously hypertensive rat; TNFα: tumor necrosis factor alpha.

## Competing interests

The authors declare that they have no competing interests.

## Authors' contributions

QS performed the experimental study, contributed to preparation of the manuscript and undertook the statistical analysis. JA, GB, HF and JCF evaluated and interpreted the data and assisted with preparation of the manuscript. MB designed the study, supervised the project, evaluated and interpreted the data, and prepared the manuscript. All authors read and approved the final manuscript
